# DSM-5 and ICD-11 as competing models of PTSD in preadolescent children exposed to a natural disaster: assessing validity and co-occurring symptomatology

**DOI:** 10.1080/20008198.2017.1310591

**Published:** 2017-04-07

**Authors:** Annette M. La Greca, BreAnne A. Danzi, Sherilynn F. Chan

**Affiliations:** ^a^ Department of Psychology, University of Miami, Coral Gables, FL, USA

**Keywords:** Preadolescent, DSM-5, ICD-11, models of PTSD, depression, anxiety

## Abstract

**Background**: Major revisions have been made to the DSM and ICD models of post-traumatic stress disorder (PTSD). However, it is not known whether these models fit children’s post-trauma responses, even though children are a vulnerable population following disasters.

**Objective**: Using data from Hurricane Ike, we examined how well trauma-exposed children’s symptoms fit the DSM-IV, DSM-5 and ICD-11 models, and whether the models varied by gender. We also evaluated whether elevated symptoms of depression and anxiety characterized children meeting PTSD criteria based on DSM-5 and ICD-11.

**Method**: Eight-months post-disaster, children (*N* = 327, 7–11 years) affected by Hurricane Ike completed measures of PTSD, anxiety and depression. Algorithms approximated a PTSD diagnosis based on DSM-5 and ICD-11 models.

**Results**: Using confirmatory factor analysis, ICD-11 had the best-fitting model, followed by DSM-IV and DSM-5. The ICD-11 model also demonstrated strong measurement invariance across gender. Analyses revealed poor overlap between DSM-5 and ICD-11, although children meeting either set of criteria reported severe PTSD symptoms. Further, children who met PTSD criteria for DSM-5, but not for ICD-11, reported significantly higher levels of depression and general anxiety than children not meeting DSM-5 criteria.

**Conclusions**: Findings support the parsimonious ICD-11 model of PTSD for trauma-exposed children, although adequate fit also was obtained for DSM-5. Use of only one model of PTSD, be it DSM-5 or ICD-11, will likely miss children with significant post-traumatic stress. DSM-5 may identify children with high levels of comorbid symptomatology, which may require additional clinical intervention.

## 1. Background

Major revisions have been made to the conceptualization of post-traumatic stress disorder (PTSD). The PTSD model provided by DSM-5 (American Psychiatric Association, 2013) differs substantially from DSM-IV (American Psychiatric Association, 1994) and ICD-11 (World Health Organization, 2017). Although these new and different conceptualizations of PTSD are intended for use with children, little is known about how well these models of PTSD fit preadolescent children’s post-trauma responses. Research on the conceptualization and diagnosis of PTSD has focused exclusively on adult, adolescent or preschool samples (Armour, 2015; Friedman, Resick, Bryant, & Brewin, 2011; Scheeringa, Zeanah, & Cohen, 2011). To our knowledge, no study has evaluated the adequacy of the DSM-5 and proposed ICD-11 models for PTSD in preadolescent children (7–11 years of age).

This is a critical gap in the literature as many children are affected by traumatic events that can result in PTSD. For example, natural disasters affect 66 million youth annually worldwide (Pronczuk & Surdu, 2008), and a substantial percentage of disaster-exposed children evidence clinical levels of PTSD (La Greca, Silverman, Lai, & Jaccard, 2010; La Greca, Silverman, Vernberg, & Prinstein, 1996; Weems et al., 2010). Furthermore, children have been identified as psychologically vulnerable in the aftermath of disasters (Dodgen, Donato, Dutta, Kelly, & La Greca et al., 2016; Norris et al., 2002). Other types of trauma (e.g. maltreatment, medical injuries) also commonly affect children and contribute to the development of PTSD (Bender, Brown, Thompson, Ferguson, & Langenderfer, 2015; Kassam-Adams, Marsac, & Cirilli, 2010). In many cases, a PTSD diagnosis has been used to identify youth in need of mental health services (Jaycox et al., 2010) or document treatment outcome (Barron, Abdallah, & Smith, 2013). Thus, it is critical to have a valid model of PTSD for identifying and treating traumatized children.

The present study evaluated the ‘fit’ between children’s post-disaster symptoms and models of PTSD based on DSM-IV, DSM-5 and ICD-11 diagnostic criteria, analyzing data from children directly exposed to a natural disaster, Hurricane Ike. Further, we examined whether other internalizing symptoms (i.e. anxiety, depression) co-occurred among children who met criteria for PTSD using DSM-5 and ICD-11 criteria. Such information would inform the conceptualization of PTSD in children and provide useful information for research and practice.

### 1.1. Appropriateness of DSM-5 and ICD-11 for preadolescents

DSM-IV used a three-factor model of PTSD that required the presence of symptoms from three clusters: re-experiencing, avoidance and arousal (American Psychiatric Association, 1994). In broadening the PTSD construct (Friedman, 2013), DSM-5 used a four-factor model with a new cognitions/mood cluster. The cognitions/mood cluster includes symptoms previously found in the DSM-IV avoidance cluster (Table 1) and new symptoms which were included to capture the complexity of different PTSD presentations observed in adults (Friedman, 2013). However, it is not clear whether these new symptoms are appropriate for preadolescents, who may lack the cognitive maturity to experience or report these symptoms. In fact, in two independent samples of preadolescents exposed to natural disasters, we found that the cognitions/mood cluster was the least frequently endorsed of the DSM-5 symptoms clusters (Danzi & La Greca, 2016).

**Table 1.** PTSD symptoms and items.

Symptom	DSM-IV	DSM-5	ICD-11	Item
Intrusive memories	RE	RE		RI #3: ‘I have upsetting thoughts, pictures, or sounds of what happened come into my mind when I do not want them to’
Nightmares	RE	RE	RE	RI #5: ‘I have dreams about the hurricane or other bad dreams’
Flashbacks	RE	RE	RE	RI #6: ‘I feel like I am back at the time when the bad thing happened, living through it again’
Psychological distress	RE	RE		RI #2: ‘When something reminds me of what happened, I get very upset, afraid or sad’
Physiological reactions	RE	RE		RI #18: ‘When something reminds me of the hurricane, I have strong feelings in my body, like my heart beats fast, my head aches, or my stomach aches’
Avoidance – internal cues	Avoid	Avoid	Avoid	RI #9: ‘I try not to talk about, think about, or have feelings about the hurricane’
Avoidance – external cues	Avoid	Avoid	Avoid	RI #17: ‘I try to stay away from people, places, or things that make me remember the hurricane’
Restricted range of affect	Avoid			RI #10 or #11: ‘I have trouble feeling happiness or love’ or ‘I have trouble feeling sadness or anger’
Shortened future	Avoid			RI #19: ‘I think that I will not live a long life’
Inability to recall trauma	Avoid	C/M		RI #15: ‘I have trouble remembering important parts of the hurricane’
Anhedonia	Avoid	C/M		RI #7: ‘I feel like staying by myself and not being with my friends’
Detachment/estrangement	Avoid	C/M		RI #8: ‘I feel alone inside and not close to other people’
Negative beliefs		C/M		CDI #5: ‘I am bad all the time’
Blame for event		C/M		RI #14: ‘I think that some part of the hurricane is my fault’
Negative emotional state		C/M		RCMAS #7: ‘I am afraid of a lot of things’
Inability to feel positive emotion		C/M		RI #10: ‘I have trouble feeling happiness or love’
Irritability/anger	Arousal	Arousal		RI #4: ‘I feel grouchy, angry or mad’
Reckless/self-destructive		Arousal		–
Hypervigilance	Arousal	Arousal	Arousal	RI #1: ‘I watch out for danger or things that I am afraid of’
Startle response	Arousal	Arousal	Arousal	RI #12: ‘I feel jumpy or startle easily, like when I hear a loud noise or when something surprises me’
Concentration	Arousal	Arousal		RI #16: ‘I have trouble concentrating or paying attention’
Insomnia	Arousal	Arousal		RI #13: ‘I have trouble going to sleep or I wake up often during the night’

Note: RE = re-experiencing, Avoid = avoidance, C/M = cognitions/mood.

Interestingly, the four-factor model of PTSD in DSM-5 was deemed unsuitable for preschool children, and separate diagnostic criteria were developed for that age group (American Psychiatric Association, 2013). However, due to insufficient research on preadolescent children, the DSM-5 adult model was extended to youth in this age range (Scheeringa et al., 2011). Yet, it is not known whether the DSM-5 model of PTSD fits the trauma responses of preadolescents as no relevant studies have been conducted with this vulnerable age group. This study used confirmatory factor analysis to examine the fit of the DSM-5 model of PTSD for trauma-exposed preadolescents.

We also examined the fit of the proposed ICD-11 model, which is still undergoing evaluation. ICD-11 takes a narrow approach to PTSD by focusing on a few core symptoms (Friedman, 2013). Specifically, it uses a three-factor model of PTSD, with symptom clusters for re-experiencing, avoidance and arousal, and requires fewer symptoms per cluster for the diagnosis than DSM-IV or DSM-5 (World Health Organization, 2017). Further, the symptoms required for the re-experiencing cluster are intended to be specific to PTSD and thus only include flashbacks and nightmares. As with DSM-5, no studies have examined whether the proposed ICD-11 model of PTSD fits the responses of trauma-exposed preadolescents.

Having two very discrepant models of PTSD is problematic for the conceptualization of the disorder (Hansen, Hyland, Armour, Shevlin, & Elklit, 2015) and may affect our understanding of its aetiology, maintenance and treatment (Elhai & Palmieri, 2011). In the present study we examined the statistical fit of the DSM-5 and ICD-11 models of PTSD, as well as the earlier DSM-IV model, for preadolescent children exposed to a natural disaster. We also evaluated measurement invariance (i.e. model equivalence) across gender, which is important to ensure that the models of PTSD are measuring the same construct in the same way for both boys and girls, and for comparing results across gender (Cheung & Rensvold, 2002).

### 1.2. Co-occurring symptomatology

We additionally examined post-traumatic stress (PTS) levels and co-occurring symptomatology of preadolescents who met criteria for PTSD based on the DSM-5 or ICD-11 models, as these are the two diagnostic systems that will be used in future. First, given DSM-5’s more complex model of PTSD (Friedman, 2013), we expected children meeting the DSM-5 criteria to report greater PTS symptom severity than those meeting criteria for ICD-11. We assessed PTS symptom severity with a widely used measure, the revised Posttraumatic Stress Disorder-Reaction Index for DSM-IV (PTSD-RI-R) because over the past two decades child disaster research has been based on the DSM-IV model of PTSD and predominantly has used this measure to assess PTS symptom severity (e.g. Felix et al., 2011; Lai, La Greca, Auslander, & Short, 2013). Thus, using PTSD-RI-R to index PTS symptom severity would facilitate comparisons between the new (DSM-5) and emerging (ICD-11) models of PTSD and prior research based on DSM-IV.

Second, we expected that symptoms of anxiety and depression would be more elevated among children meeting criteria for the DSM-5 versus the ICD-11 model. Symptoms of anxiety and depression commonly co-occur with PTSD in children and are predictive of poorer post-traumatic recovery (Felix et al., 2011; Goenjian et al., 2001; Lai et al., 2013; Weems et al., 2010). DSM-5 PTSD includes several symptoms that overlap with depression (e.g. anhedonia, insomnia, excessive guilt or blame) or reflect anxiety (e.g. physiological reactions, negative emotional state) (Table 1). Understanding the presence of co-occurring anxiety and depression among children meeting PTSD criteria might inform the conceptualization of PTSD in children, which is important for researchers as well as those who provide clinical services to trauma-exposed children. For example, if DSM-5 is more likely than ICD-11 to identify children with elevated symptoms of depression, mental health providers using DSM-5 diagnostic criteria may need to be prepared to treat depressive symptoms, as well as PTSD, in traumatized youth.

## 2. Objective

We evaluated the statistical fit of the DSM-5 and ICD-11 models of PTSD, as well as associated PTS symptom severity and co-occurring symptomatology, in preadolescents exposed to Hurricane Ike, a destructive natural disaster. Ike struck Galveston in Texas, U.S.A., in September 2008, taking 103 lives and causing US$24.9 billion in damage due to the storm surge, widespread flooding and sustained winds of 175 km/h. Hundreds of families were displaced from their homes and all schools were closed at least temporarily (Berg, 2008).

One advantage of using a disaster to test models of PTSD is that all children were exposed to the same potentially traumatic event and were assessed at the same time point post-trauma (in this case, 8 months post-disaster). By 8–10 months post-disaster, children with elevated symptoms of PTSD are likely to have a chronic course of symptomatology (La Greca et al., 2013b).

Using this sample of trauma-exposed preadolescents, we evaluated three models of PTSD for goodness of fit: DSM-IV, DSM-5 and ICD-11. We also evaluated the models’ measurement invariance across gender. Finally, we compared children who likely met criteria for DSM-5 or ICD-11 on their levels of PTS symptom severity and co-occurring symptoms of anxiety and depression.

## 3. Method

### 3.1. Participants

Participants were 327 children (7–11 years; mean* = *8.73 years, SD* = *0.98 years; 52% girls; grades 2–4) who were ethnically/racially diverse (36% Hispanic, 27% White, 19% Black, 14% Other, 4% Asian), reflecting the school district’s diversity (New American Foundations, 2012). All children from all six elementary schools in the Galveston Independent School District (GISD) were recruited in May 2009. Two schools were so badly damaged that they were closed for a year or more; children who attended these schools were reassigned to a habitable school. In 2008, 65.9% of the students were eligible for free or reduced-price lunch (an index of economic disadvantage), which rose to 76.5% in 2009 (New America Network, 2012).

Children reported high levels of disaster exposure. Specifically, 95% of the sample reported at least one life-threatening event (e.g. seeing someone get hurt, thinking they might die) or at least one event reflecting immediate disaster-related loss and disruption. The most common disaster-related event was having one’s home damaged or destroyed (53%).

### 3.2. Procedures

The relevant university institutional review boards and the GISD approved the study. Parental consent forms were distributed to all children in grades 2–4, and returned to classroom teachers. Active parental (or legal guardian) consent and child assent were required for participation. Of the forms that were returned, consent was provided for 340 students (69%). This represents about 35% of the eligible youth; this response rate is similar to other studies of children post-disaster (e.g. Moore & Varela, 2010). Attrition was primarily due to children being absent the days of testing.

Children were informed that the study was about reactions to Hurricane Ike and how children think and feel after a hurricane. Group testing occurred in the schools, under close supervision of multiple research assistants. Children were instructed to look only at their own surveys, were seated apart from their classmates and were not allowed to talk during the testing. Children were instructed to think about the hurricane when completing all the questionnaires. Questions were read aloud to facilitate children’s understanding (for details, see La Greca, Lai, Joormann, Auslander, & Short, 2013a).

### 3.3. Measures

All measures were child report. A demographic questionnaire asked about gender, ethnicity and age.

#### 3.3.1. PTSD

The PTSD-RI-R (Steinberg, Brymer, Decker, & Pynoos, 2004) assessed PTSD symptoms based on DSM-IV. It contains 22 items rated: 0 = None of the time, 2 = Some of the time and 4 = Most of the time. This widely used measure has good convergent validity, internal consistency and test–retest reliability (Steinberg et al., 2004, 2013).

For analyses of PTS symptom severity, the 17 items included in the DSM-IV scoring of the measure were used and children’s ratings were summed to obtain a score for symptom severity (possible range = 0–68). Total PTSD-RI-R scores of 38 or higher reflect clinical levels of PTSD (Steinberg et al., 2004).

The PTSD-RI-R items also were used as indicators of PTSD symptoms for the DSM-IV, DSM-5 and ICD-11 diagnostic algorithms. Symptoms were counted as present if the child reported experiencing the symptom ‘most of the time’. Items assessed all the symptoms included in DSM-IV and ICD-11 models of PTSD, and almost all the symptoms in the DSM-5 model. Specifically for DSM-IV, all 17 PTSD symptoms were assessed by 18 of the items^1^ on the PTSD-RI-R following standard scoring procedures (Steinberg et al., 2004).

The methodology used for assessing DSM-5 and ICD-11 PTSD has been published elsewhere (Danzi & La Greca, 2016). Briefly, for DSM-5, 17 of the 20 symptoms were represented by items on the PTSD-RI-R (Table 1). Because the study was conducted prior to the publication of DSM-5, three symptoms new to the diagnosis were not represented: negative beliefs, negative emotional state and reckless/self-destructive behaviour. Consequently, four independent coders (clinical psychology doctoral students) rated items from available measures on the extent to which they approximated these symptoms. Face validity and shared wording with DSM-5 text were emphasized. One Children’s Depression Inventory item (*I am bad*) was selected to approximate negative beliefs. This item is nearly identical to the one added to the PTSD-RI  for DSM-5 to assess negative beliefs (*I have thoughts like ‘I am bad’*; Pynoos & Steinberg, 2014), although it is not as broad as the DSM-5 description. Children who endorsed the most severe form of this item were considered to meet criterion for negative beliefs. One item from the Revised-Children’s Manifest Anxiety Scale (*I am afraid of a lot of things*) was selected to approximate negative emotional state, consistent with (but more narrow than) the DSM-5 criterion D4: ‘Persistent negative emotional state (e.g. fear, horror, anger, guilt, or shame)’ (American Psychiatric Association, 2013). Children who endorsed this item were considered to meet criterion for negative emotional state. Finally, no items had adequate face validity to represent reckless/self-destructive behaviour, which is challenging to assess in preadolescents as it was intended to reflect behaviours, such as reckless driving and risky sexual behaviours (Friedman et al., 2011).

Finally, for ICD-11, six items on the PTSD-RI-R represented the relevant symptoms. Children needed to endorse only one symptom per cluster to meet criteria for the ICD-11 definition (Table 1).

In addition to symptom criteria, children had to report disaster-related impairment to ‘meet criteria’ for PTSD. Impairment was assessed by four items adapted from the Anxiety Disorders Interview Schedule for DSM-IV; Child Version (ADIS-C; Silverman & Albano, 1996). Response options (0 = None to 3 = Very much) were summed for a possible range of 0–12. Impairment was indicated using a cut-off score of 3 (Danzi & La Greca, 2016).

#### 3.3.2. Anxiety and depression

Anxiety symptoms were assessed with the Revised-Children’s Manifest Anxiety Scale (RCMAS) that contains 28 items (rated *Yes* or *No)*. The RCMAS is among the most widely used measures of child anxiety and has extensive psychometric support (Reynolds & Richmond, 1985).

The Children’s Depression Inventory (CDI) is a widely used measure of behavioural, cognitive and affective symptoms of depression (Kovacs, 1981). It includes 27 items with three levels of severity for each item; one item regarding suicidal ideation was removed due to ethical concerns. For each item, children selected the statement that best described them; scores were summed across items.

### 3.4. Analyses

Preliminary analyses (using SPSS v.22) examined outliers and normality. Missingness was handled with multiple imputation for the preliminary analyses and analysis of variance (ANOVA). No substantial outliers, skewness or kurtosis were observed. Means and standard deviations (SD) were computed, and bivariate correlations were conducted for PTS symptom severity, depression and anxiety.

The PTSD models were examined using confirmatory factor analysis (CFA) (Mplus v.7.31; Muthén & Muthén, 1998–2012). Only children who reported direct trauma exposure were included (*n *= 310). This sample size (> 200–300) is considered desirable (O’Rourke & Hatcher, 2013). Missing data were handled with full information maximum likelihood (Kline, 2011). Based on the algorithms, latent variables for the re-experiencing, avoidance, arousal and cognitions/mood clusters were created using items from the PTSD-RI-R, CDI and RCMAS as indicators. PTSD-RI-R items were used as continuous measures and CDI and RCMAS items were recoded to match the PTSD-RI-R coding. Model fit was evaluated using these guidelines: Bentler Comparative Fit Index (CFI) > .90, Tucker Lewis Index (TLI) > .90, root mean square error of approximation (RMSEA) < .06, and standardized root mean square residual (SRMR) < .08 (Hu & Bentler, 1999; Kline, 2011). The Akaike information criterion (AIC) and Bayesian information criterion (BIC) also compared the relative fit of different models, with lower values indicating a better trade-off between model fit and complexity (Kline, 2011; Van de Schoot, Lugtig, & Hox, 2012). Measurement invariance was tested across gender using Mplus (v.7.1 Mplus addendum). We were unable to compare directly the PTSD models using statistical procedures because the models were not nested.

To evaluate the distress levels of children meeting DSM-5 and ICD-11 criteria for PTSD, we computed the percentages of children meeting criteria for probable PTSD across the diagnostic systems (DSM-5 only, ICD-11 only, both DSM-5 and ICD-11, neither). Using SPSS v.22, a two-way (DSM-5 present/absent; ICD-11 present/absent) ANOVA evaluated PTS symptom severity. Multivariate two-way ANOVAs evaluated co-occurring symptomatology (anxiety, depression). Gender, age and minority status were controlled in these analyses. Girls reported more PTS severity (*p *< .05) and symptoms of anxiety (*p *< .001) than boys. Younger children and those from minority backgrounds reported more symptoms of PTS, anxiety and depression than older youth and non-minorities, respectively (all *p*’s < .01).

## 4. Results

Overall, children reported moderate PTS symptom severity (PTSD-RI-R, mean = 24.79; SD* *= 14.64; based on DSM-IV scoring; Steinberg et al., 2004). Children’s depression (CDI, mean = 11.99, SD* *= 7.68) and anxiety (RCMAS, mean = 12.40, SD* *= 7.69) were in the average range. Depression levels were comparable with other post-disaster studies (mean *= *11.23, 6 months post-earthquake among children in grades 4–6; Kolaitis et al., 2003), as were anxiety levels (mean *= *10.27, 3 months post-hurricane among children in grades 3–5; La Greca et al., 2013b). Finally, PTS symptom severity was correlated with symptoms of depression (*r *= .58, *p *< .001) and anxiety (*r *= .72, *p *< .001). Symptoms of depression and anxiety also were correlated (*r *= .65, *p *< .001).

### 4.1. PTSD model fit

Among trauma-exposed children, for the three-factor model of DSM-IV, CFA fit indices revealed an acceptable fit (Table 2); factor loadings ranged from .55 to .65 for re-experiencing, from .42 to .58 for avoidance, and from .38 to .57 for arousal. Only one item had a loading below .40 (‘startle response’ from the arousal cluster).

**Table 2.** Model fit statistics from confirmatory factor analysis for the three DSM models and ICD-11.

PTSD Model	*χ*^2^	d.f.	*p*	CFI	TLI	RMSEA (90% CI)	SRMR	AIC	BIC
DSM-IV	180.63	116	.0001	0.940	0.930	.042 (.030–.054)	0.046	18,419.35	18,621.13
DSM-5	205.64	146	.0008	0.947	0.938	.036 (.024–.048)	0.046	20,384.59	20,619.59
ICD-11	6.85	6	.33	0.997	0.991	.021 (.000–.079)	0.022	6723.46	6801.92

Note: *χ* ^2^ = chi-square goodness of fit statistics; d.f. = degrees of freedom; CFI = Comparative Fit Index; TLI = Tucker Lewis Index; RMSEA (90% CI) = root mean square error of approximation with 90% confidence intervals; SRMR = standardized root mean square residual; AIC = Akaike information criterion; BIC = Bayesian information criterion.

**Figure 1.**
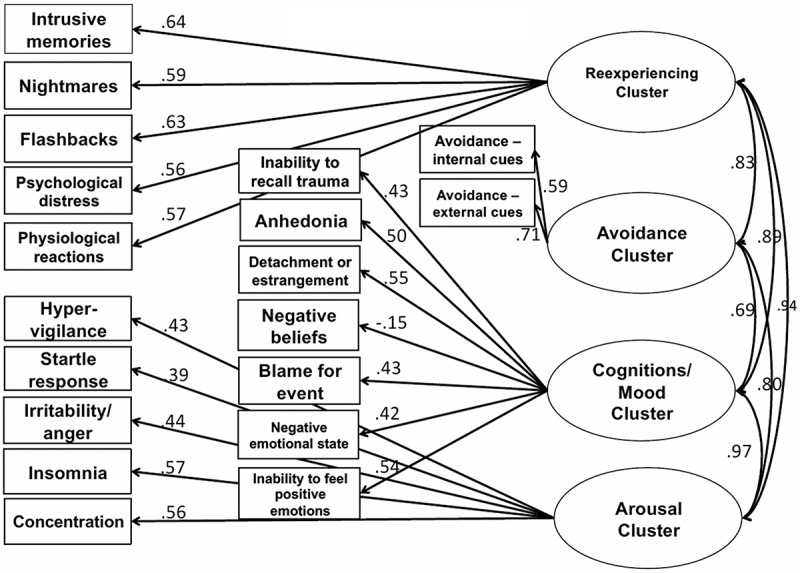
Model for DSM-5.

Similarly, the four-factor model of DSM-5 had an acceptable fit (Table 2 and Figure 1). Factor loadings ranged from .56 to .64 for re-experiencing, from .59 to .71 for avoidance, and from .39 to .57 for arousal (‘startle response’ again had a low loading). For the new cognitions/mood cluster, factor loadings ranged from –.15 to .55, with one item below .40 (‘negative beliefs’). When this item was removed, the CFA fit statistics were very similar. Given these negligible differences, we retained the initial DSM-5 model as it more closely approximates the items used in the DSM-5 diagnostic model.

**Figure 2.**
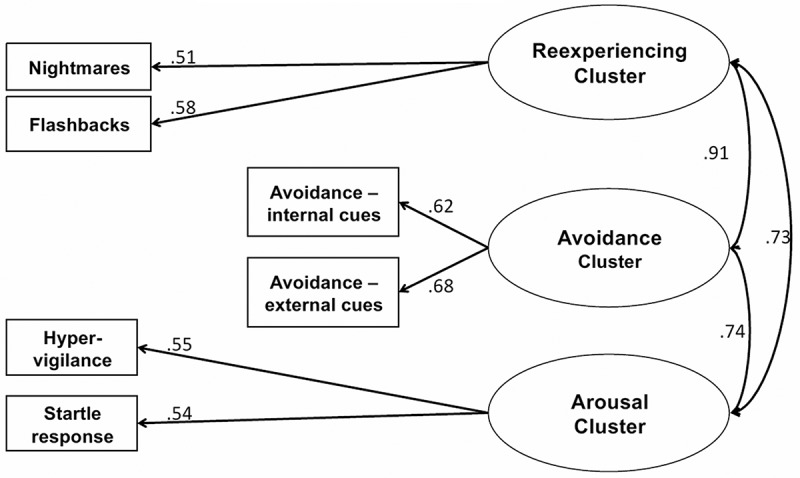
Model for ICD-11.

For ICD-11, we obtained an excellent fit for the three-factor model (Table 2 and Figure 2). Factor loadings ranged from .51 to .68 across the clusters. Although we could not directly compare the ICD and DSM models, the fit statistics appeared better for ICD-11 compared with DSM-IV and DSM-5. Nevertheless, the fit for DSM-5 also was acceptable and similar to the fit for DSM-IV.

Next, we evaluated whether each model fitted equally well for boys and girls by testing for invariance across gender. In a stepwise manner, we tested for configural invariance (i.e. whether the factor structure holds across groups; no parameters constrained), metric invariance (i.e. whether factor loadings are equal across groups), and scalar invariance (i.e. whether factor loadings and intercepts are equal across groups). We compared the metric models to the configural models and then the scalar models to the metric models.

The fit statistics for tests of gender invariance (Table 3) revealed that the configural, metric and scalar models for DSM-IV and DSM-5 showed acceptable fit (CFI > .90, RMSEA <.06 and SRMR <.08). The model for ICD-11 showed excellent fit (i.e. additionally, there was a non-significant chi-square). For all three PTSD models, comparisons between the fit of the metric and configural models were not significantly different (all *p’*s > .50). This indicates that constraining factor loadings across groups does not significantly worsen model fit, thus establishing metric invariance across gender. Further, for ICD-11, the fit of the scalar model was not significantly different from the metric model (*χ* ^2^(3) = 3.22, *p *= .36), revealing scalar (i.e. strong) measurement invariance across gender for ICD-11. In contrast, the scalar model fit significantly worse than the metric model for DSM-IV (*χ* ^2^(14) = 26.39, *p *= .02) and DSM-IV (*χ* ^2^(15) = 37.82, *p *= .001). Thus, only metric (i.e. weak) invariance was obtained across gender for DSM-IV and DSM-5; factor loadings but not the intercepts were similar across boys and girls.

**Table 3.** Tests of gender invariance for DSM-IV, DSM-5 and ICD-11 models.

Model	*χ*^2^	d.f.	*p*	CFI	TLI	RMSEA (90% CI)	SRMR	AIC	BIC
***DSM-IV***									
Configural	279.76	232	.02	0.948	0.939	.036 (.016–.051)	0.061	18,451.64	18,855.19
Metric	288.64	246	.03	0.954	0.949	.033 (.011–.048)	0.062	18,431.17	18,782.41
Scalar	313.98	260	.01	0.941	0.939	.037 (.018–.050)	0.065	18,429.96	18,728.89
									
***DSM-5***									
Configural	350.47	292	.01	0.940	0.930	.036 (.019–.049)	0.061	20,397.83	20,867.82
Metric	363.01	307	.02	0.943	0.936	.034 (.016–.048)	0.067	20,379.40	20,793.44
Scalar	399.58	322	.002	0.922	0.917	.039 (.025–.051)	0.069	20,387.35	20,745.44
									
***ICD-11***									
Configural	10.64	12	.56	1.000	1.017	.000 (.000–.074)	0.030	6732.30	6889.24
Metric	12.99	15	.61	1.000	1.020	.000 (.000–.066)	0.036	6728.74	6874.47
Scalar	16.18	18	.58	1.000	1.015	.000 (.000–.064)	0.041	6726.07	6860.59

Note: *χ* ^2^ = chi-square goodness of fit statistics; d.f. = degrees of freedom; CFI = Comparative Fit Index; TLI = Tucker Lewis Index; RMSEA (90% CI) = root mean square error of approximation with 90% confidence intervals; SRMR = standardized square root mean residual; AIC = Akaike information criterion; BIC = Bayesian information criterion.

### 4.2. Co-occurring symptomatology

Using algorithms described previously, we identified 16.2% of the sample as likely meeting either DSM-5 or ICD-11 criteria for PTSD (Table 4). Specifically, 5.2% of the children met criteria for DSM-5 only, 3.7% met criteria for ICD-11 only and 7.3% met criteria for both. Overlap between the models was poor, as only 45% of those identified as meeting criteria for PTSD fit the criteria for both DSM-5 and ICD-11.

**Table 4.** Co-occurring symptomatology of children likely meeting criteria (including impairment) for DSM-5 or ICD-11 models of PTSD.

	Models of PTSD				
	DSM-5 only	ICD-11 only	DSM-5 plus ICD-11	No PTSD	Significant effects
Number of children (%)	17 (5.2%)	12 (3.7%)	24 (7.3%)	274 (83.8%)	
***Demographics***					
Gender (% girls)	53%	67%	48%	51%	n.s.
Age (years)	8.59 (1.00)	8.52 (1.07)	8.38 (0.99)	8.78 (0.97)	n.s.
Minority (%)	88%	75%	87%	70%	n.s.
PTSD symptom severity^a^	44.12 (5.87)	38.13 (6.68)	50.07 (8.07)	20.84 (12.03)	DSM-5: *F*(1) = 49.44, *p* < .001 ICD-11: *F*(1) = 21.13, *p* < .001 Interaction: *F*(1) = 4.62, *p* < .05
***Co-occurringsymptoms***^a^					
Anxiety (RCMAS)	19.37 (6.88)	16.86 (4.81)	19.90 (5.15)	11.12 (7.38)	DSM-5: *F*(1) = 13.94, *p* < .001
Depression (CDI)	17.91 (5.97)	15.43 (5.48)	19.54 (5.83)	10.78 (7.41)	DSM-5: *F*(1) = 10.58, *p* < .01

Note: ^a^Controlling for gender, age and minority status in the analyses; *N* = 327.

Table 4 also contains the symptom levels reported by the children and the ANOVA results. Two key findings emerged. First, for PTS symptom severity, significant main effects were observed, and a significant interaction. Children who met criteria for either DSM-5 or ICD-11 reported higher PTS symptom severity than those who met neither criteria. Further, the interaction revealed that children meeting criteria for DSM-5-only reported greater PTS symptom severity than children meeting criteria for ICD-11-only (*F*(1) = 4.62, *p *< .05). On average, PTS symptom levels were in the severe range for children meeting criteria for ICD-11, DSM-5, or DSM-5 plus ICD-11 (based on cut-offs reported by Steinberg et al., 2004).

Second, for co-occurring symptoms of anxiety and depression, a significant main effect was observed for DSM-5 only (Table 4). Specifically, children who met criteria for DSM-5 reported higher levels of general anxiety and depression than did those not meeting DSM-5 criteria. On average, these symptom levels were 1 SD above those reported by children who did not meet criteria for PTSD. No significant elevations in anxiety or depression were apparent for those meeting criteria for ICD-11.

## 5. Discussion

The concept of PTSD in children is relatively new, having been introduced in the 1980s (Brewin, 2016), although it was primarily considered to be an adult disorder. Since that time, and largely based on research conducted with DSM-IV, it is apparent that PTSD does occur in children, and that children are a vulnerable population in the aftermath of traumatic events such as disasters (Norris et al., 2002). However, the conceptualization of PTSD in children still remains in question. A precise understanding of what constitutes PTSD in children is critical for guiding future research and clinical practice. This study is the first to investigate the latent structure of PTSD with the DSM-5 and proposed ICD-11 models among preadolescent children exposed to a traumatic event.

### 5.1. PTSD model fit and conceptualization

The models of PTSD reflected in DSM-5 and ICD-11 both fit children’s post-disaster responses. Analyses supported the latent structure of PTSD as reflected in the DSM-5 and ICD-11 models, as well as in the earlier DSM-IV model.

Further, we obtained support for strong measurement invariance across gender for the ICD-11 model, indicating that this model performs equally well for boys and girls and that the (latent) means can be compared across gender. For DSM-IV and DSM-5, the underlying model of PTSD was similar for boys and girls, but due to weak measurement invariance across gender, latent means between boys and girls cannot reasonably be compared.

Our findings are consistent with recent work with adults (e.g. Hansen et al., 2015), which found that the latent structure of PTSD is simpler than that suggested by DSM-5. One appeal of the ICD-11 model is its clinical utility, which contributes to ease of diagnosis and treatment management (Cloitre, Garvert, Brewin, Bryant, & Maercker, 2013). The clinical utility of ICD-11 could be advantageous for rapidly identifying and treating trauma-exposed children. Nevertheless, we obtained an adequate fit with both the DSM-5 and ICD-11 models, and both models identified youth with significantly elevated levels of PTS symptom severity compared with children not meeting criteria for PTSD. Evaluating how well the DSM-5 and ICD-11 criteria identify children with more persistent PTSD represents an important future direction.

Thus, at least for now, the debate remains open as to which is the preferred model for capturing PTSD in preadolescents. Further research evaluating conceptual models of PTSD in children exposed to disasters and other potentially traumatic events will be essential to resolving this issue. Alternative models for PTSD have been investigated in younger (Scheeringa, Myers, Putnam, & Zeanah, 2012) and older (Liu, Wang, Cao, Qing, & Armour, 2016) youth. In particular, future research might evaluate the utility of extending the DSM-5 preschool criteria to preadolescents. Furthermore, work with trauma-exposed adults (Cloitre et al., 2013) also supports the value of organizing the traumatic stress diagnosis into distinct types, PTSD and complex PTSD, a line of research that could be extended to preadolescents. Investigations of PTSD in children exposed to other types of potentially traumatic events (e.g. medical injury, severe abuse) also are needed.

### 5.2. Difference in the conceptual models

We found poor overlap between the DSM-5 and ICD-11 models, as less than half of those identified as meeting criteria for PTSD fit the criteria for both DSM-5 and ICD-11. This discrepancy is concerning, as a clinical diagnosis may be required for children to receive mental health services. The exclusive use of only one model of PTSD, be it DSM-5 or ICD-11, may miss a substantial percentage of youth who report significant elevations in post-traumatic stress. Yet, our findings confirmed that *both* the DSM-5 and ICD-11 models (including impairment) identified children with significant PTS symptom severity. The results are consistent with studies of adults that found a substantial portion of those diagnosed with PTSD met criteria for one but not the other set of criteria (e.g. O’Donnell et al., 2014).

We additionally found that children meeting criteria for DSM-5 (but not ICD-11) reported significant elevations in symptoms of anxiety and depression. Similarly, in adults, DSM-5 shows higher rates of comorbidity with depression than does ICD-11 (O’Donnell et al., 2014). In fact, DSM-5 criteria for PTSD have been criticized for having too much symptom overlap with depression (Brewin, Lanius, Novac, Schnyder, & Galea, 2009). Our findings indicate that the DSM-5 PTSD criteria may identify children with substantial symptoms of both depression and anxiety, in addition to PTSD. Notably, our study did not address the ICD-11 definition for complex PTSD (Cloitre et al., 2013), which is intended to identify those with substantial comorbidities. Studies that evaluate definitions of complex PTSD in children are needed.

From a research perspective, disentangling PTSD from commonly comorbid conditions is desirable as it enhances the ability to study risk mechanisms that are specific to PTSD. At present, however, our findings suggest that the DSM-5 model of PTSD may identify children with more persistent and chronic PTSD. Earlier findings revealed that trauma-exposed children with co-occurring elevations in PTSD and general anxiety (La Greca et al., 2013b) or depression (Lai et al., 2013) have poorer long-term outcomes and are more likely to report chronic PTSD. Traumatized children with co-occurring depressive symptoms also appear to be more resistant to treatment (Jaycox et al., 2010). Thus, overall, children meeting DSM-5 criteria for PTSD might need complex interventions that address symptoms of anxiety or depression, in addition to PTSD.

### 5.3. Limitations and conclusions

Despite multiple strengths, the study’s limitations should be considered. First, our analyses were based on child report. Obtaining reports directly from children is essential, as parents are poor reporters of PTSD symptoms in their children (Stover, Hahn, Im, & Berkowitz, 2010). Further, as is typical after community-wide disasters (Felix et al., 2011; Weems et al., 2010), we used self-report measures because it was not feasible to administer diagnostic interviews to large numbers of affected youth. However, future studies that incorporate structured clinical interviews would be desirable.

Second, because data were collected prior to the release of DSM-5, we used items from other measures to approximate two symptoms (negative emotional state, negative beliefs) that may not have fully captured the broader constructs intended by DSM-5. A third DSM-5 symptom, reckless/self-destructive behaviour, could not be assessed with available measures. Consequently, we may have underestimated the prevalence of DSM-5 PTSD. However, reckless/self-destructive behaviours (e.g. reckless driving, risky sexual behaviours) were added to the DSM-5 criteria based on observations of adults (Friedman et al., 2011) and are infrequent among preadolescents. For example, the US Centers for Disease Control and Prevention (CDC) does not even track such risk behaviours (e.g. alcohol/drug use, risky sex, suicide) until youth are 12 years old.^2^ Future investigators similarly may find this DSM-5 symptom challenging to assess with preadolescents.

Third, our items assessing re-experiencing symptoms were broader than the current ICD-11 definition, which indicates that such reactions be accompanied by strong physical sensations or feelings of being immersed in the emotions that were experienced during the event (World Health Organization, 2017). Using this more restrictive definition of re-experiencing likely would have lowered the prevalence rate for ICD-11 and widened the gap between DSM-5 and ICD-11 cases. ICD-11 developers might consider using a broader definition for re-experiencing, at least with children who could have difficulty reporting such complex reactions.

Finally, we focused on children who experienced a natural disaster. Thus, the current findings may not generalize to children exposed to other potentially traumatic events.

Although requiring further replication, our findings nonetheless have important implications for research and practice with children. Both models of PTSD identified youth with elevated symptoms of PTSD but also missed youth with significant elevations in post-traumatic stress. Until further clarity is achieved, assessing child symptoms that capture elements of both DSM-5 and ICD-11 models of PTSD may be essential.

## Notes

1. Because the DSM-IV Criterion C6 (restricted range of affect) was assessed by two items, the more elevated of the items determined the presence of that symptom.

2. See https://www.cdc.gov/parents/teens/risk_behaviors.html/.
